# Estimation of time of HIV seroconversion using a modified CD4 depletion model

**DOI:** 10.1371/journal.pone.0246135

**Published:** 2021-02-12

**Authors:** Viviane D. Lima, Lu Wang, Paul Sereda, Taylor McLinden, Rolando Barrios, Julio S. G. Montaner

**Affiliations:** 1 British Columbia Centre for Excellence in HIV/AIDS, Vancouver, Canada; 2 Division of Infectious Diseases, Department of Medicine, Faculty of Medicine, University of British Columbia, Vancouver, Canada; 3 Vancouver Coastal Health, Vancouver, Canada; Ohio State University, UNITED STATES

## Abstract

**Introduction:**

Several methods have been proposed to estimate the time of HIV seroconversion, including those based on CD4 cell depletion models. However, previous models have failed to consider the heterogeneity that exists in CD4 trajectories among different sub-populations. Our objective was to estimate the time from HIV seroconversion relative to the HIV diagnosis date in a population-based cohort of people living with HIV (PLWH) in the province of British Columbia, Canada.

**Methods:**

We used linked administrative and clinical data from the British Columbia Seek and Treat for Optimal Prevention of HIV/AIDS (STOP HIV/AIDS) cohort, which contains longitudinal individual-level data on all PLWH ever diagnosed in the province. Eligible participants were aged ≥18 years and diagnosed with HIV between 1989 and 2013. The outcome was pre-antiretroviral treatment CD4 cell count measurements assessed every six months. Models were stratified by age and stage of HIV infection at diagnosis. Several explanatory variables were considered including longitudinal viral load measurements. Longitudinal CD4, square root transformed, was modeled via a non-linear mixed effects model; time was modeled using an exponential decay function. We assumed a Gaussian distribution (identity link), an AR(1) correlation structure, and a random intercept and slope for the longitudinal viral load measurements. Due to the population variation in CD4 count among uninfected individuals, we assumed 500 to 1500 cells/mm^3^ as the normal range when estimating the time of HIV seroconversion.

**Results:**

Longitudinal data on 1,253 individuals were analysed: 80% male, 33% White, and the median age at diagnosis was 38 years (25^th^-75^th^ percentile [Q_1_-Q_3_], 31 to 45). CD4 decay differed by stage of infection at diagnosis and age, with those ≥50 years in Stages 1 and 2 experiencing a faster decline in CD4 over time. The median duration of infection from seroconversion until HIV diagnosis was 6.9 (Q_1_-Q_3_, 3.9 to 10.1) years.

**Conclusions:**

Considering the heterogeneity that exists in individual CD4 cell trajectories in a population, we presented a methodology that only relies on routinely collected HIV-related data, which can be further extended to estimate other epidemic measures.

## Introduction

In Canada, as in other high-resource countries, people living with HIV (PLWH) are living longer than ever before due to the success of antiretroviral treatment (ART) [1, 2]. In addition to preventing morbidity and mortality due to HIV, ART has also been shown to stop HIV transmission [3–5]. In order to assess whether countries are moving towards HIV epidemic control [6], it is important to find reliable methods that can be used to estimate important epidemic measures of morbidity, including the time that it takes for an individual infected with HIV to be diagnosed. This information is key to enhancing HIV testing programs and linkage to care, and, therefore, to decreasing health disparities across population subgroups.

The method that we propose in this study is based on CD4 cell count (hereafter referred to CD4) depletion [7–11]. This methodology is flexible enough to consider inherent heterogeneities that exist in a population and it can be further applied to estimate other epidemic measures such as HIV incidence and prevalence. This model is based on longitudinal individual-level information on biomarkers (e.g., CD4, HIV viral load) and demographic factors (e.g., age, sex), and it can be extended to include information on determinants of health (e.g., biological, behaviour and environmental factors) and other factors that are known to influence the natural history of HIV [11]. Therefore, we propose to estimate the duration of infection from HIV seroconversion until diagnosis, age at seroconversion, and year of seroconversion in a population-based cohort of PLWH in the province of British Columbia (BC), Canada using a CD4 depletion model while considering different demographic, clinical and behavioural variables.

## Materials and methods

### Data source and study population

In BC, through the provincial Seek and Treat for Optimal Prevention of HIV/AIDS (STOP HIV/AIDS) population-based retrospective cohort, we have access to longitudinal individual-level data on all PLWH since their date of HIV diagnosis [12, 13]. The STOP HIV/AIDS cohort is based on a data linkage from the BC Centre for Excellence in HIV/AIDS Drug Treatment Program (DTP) clinical registry and several administrative databases containing health information on all diagnosed PLWH (regardless of whether they are accessing ART in BC or not) [13–18]. Since 1992, BC residents living with HIV have had access to centralized and publicly funded ART (through the DTP) and specialized HIV laboratory monitoring, in accordance with the BC Centre for Excellence in HIV/AIDS HIV therapeutic guidelines [19]. Data captured in the STOP HIV/AIDS cohort includes socio-demographic (e.g., sex, age, ethnicity, geographic location of residence), clinical (e.g., CD4, plasma HIV viral load, AIDS-defining illness, mortality), healthcare utilization (e.g., hospitalization, non-ART prescriptions, physician visits) and treatment variables (e.g., antiretroviral regimen information, date of ART initiation). The databases included in the STOP HIV/AIDS cohort, along with their corresponding data capture are comprehensively detailed in the Supplement.

In our study, eligible individuals were aged ≥18 years at HIV diagnosis, which happened between 1989 and 2013, and they were followed until they started ART treatment between 1996 and 2015, the last contact date with the provincial healthcare system (e.g., a physician visit, hospitalization, laboratory test), the date of death, study end, or the date in which they moved out of BC. Additionally, individuals were required to have at least two measurements of CD4 and viral load during follow-up. All viral load measurements in BC are centrally done at the St. Paul’s Hospital virology laboratory. Since the quantification range of viral load assays has evolved over time, for analytical purposes, we truncated our measurements to range from <500 (coded as 499) to >100,000 (coded as 100,010) copies/mL [20–23]. CD4 is measured by flow cytometry, followed by fluorescent monoclonal antibody analysis (Beckman Coulter, Inc., Mississauga, Ontario, Canada). The CD4 data are measured at different laboratories across BC; however, we capture >85% of all CD4 tests done in BC in our database. In addition, we removed CD4 values that were outside the normal range for this biomarker (i.e., >1500 cells/mm^3^) [24].

### Statistical analysis

CD4 and viral load measurements were obtained every six months from the time since HIV diagnosis until ART initiation, in order to model the CD4 depletion trajectory in our study. Longitudinal CD4, square root transformed, was modeled via a non-linear mixed effects model; follow-up time was modeled using an exponential decay function [25], assuming a Gaussian distribution (identity link), an AR(1) correlation structure, and a random intercept and slope for the longitudinal viral load measurements:

$$\sqrt{CD4_{i,t}}∼\gamma e^{-R*t_{i}}+\alpha_{0,i}+\alpha_{1,i}log_{10}{\left(ViralLoad\right)}_{i,t}+\beta_{1}x_{1,i}+\cdots +\beta_{n}x_{n,i}+\varepsilon_{i,t}$$

where *t* represents each of the 6-month intervals; *i* represents each individual in the study; *ε*_*i*_ is the random error distributed as ***N***(**0**,**K**_*i*_), where **K** is the covariance matrix and independent of *α*_0,*i*_ and *α*_1,*i*_, which are the random intercept and slope that varies across individuals, and they follow a bivariable normal distribution $N\left[\left(\alpha_{0},\alpha_{1}\right),\left(\sigma_{\alpha_{0}},\sigma_{\alpha_{1}}\right),{Ρ}_{\sigma_{\alpha_{0}},\sigma_{\alpha_{1}}}\right]$, where **P** is the covariance matrix. We also assumed an AR(1) correlation structure for the error term, and an unstructured variance-covariance matrix for the bivariable normal distribution. The coefficients *β*_1_, ⋯ *β*_n_ are for the fixed explanatory variables *x*_1_, ⋯ *x*_2_. The exponential decay function was modeled via the term *γe*^-R*t^, where *γ* and *R* are coefficients in this function. If the exponential decay function was not justified based on model selection, we fitted instead a linear mixed effects model. Model selection was based on a published method by our group, based on Akaike Information Criterion and significance level [26, 27]. Goodness-of-fit was based on residual diagnostic plots (Supplement). Analyses were performed in R© version 3.6.3 using the libraries nlme, mgcv, and ggplot2.

As the CD4 trajectories are expected to be different across population subgroups, the models were stratified by age (<50 versus ≥50 years) and stage of HIV infection at diagnosis (Stage 1: CD4≥500, Stage 2a: CD4 350–499, Stages 2b&3: CD4 <350 cells/mm^3^) [11]. In the HIV field, we usually define older PLWH as those ≥50 years [28–30]. In addition, studies have shown that age is an important factor when estimating HIV disease progression using CD4 depletion models [11, 31]. Thus, based on the heterogeneity in CD4 trajectories associated with age, we decided to conduct an age-stratified analysis (<50 versus ≥50 years). Note that we did not model individuals whose laboratory criteria indicated acute or recent HIV infection [32]. This decision was made “a priori” based on the established fact that the CD4 during acute HIV infection experience a sharp temporary decline, and estimating time of infection using a methodology based on CD4 would not yield valid results [11, 33]. Explanatory variables included longitudinal viral load measurements (in log_10_), sex (female/male), year of HIV diagnosis (<1996, 1996–1999, 2000–2003, 2004–2007, 2008–2013), ethnicity (White, non-White, unknown), HIV transmission risk group (gay, bisexual and other men who have sex with men [gbMSM], people who have ever injected drugs [PWID], GBMSM/PWID, heterosexual, other/unknown), AIDS at HIV diagnosis, and follow-up time (years) from HIV diagnosis until ART initiation. Note that we do not have individual-level information on HIV subtype in the STOP HIV/AIDS cohort, but a recent publication using the DTP data estimated that 86% of PLWH in BC have HIV subtype B [34].

**Fig 1:**
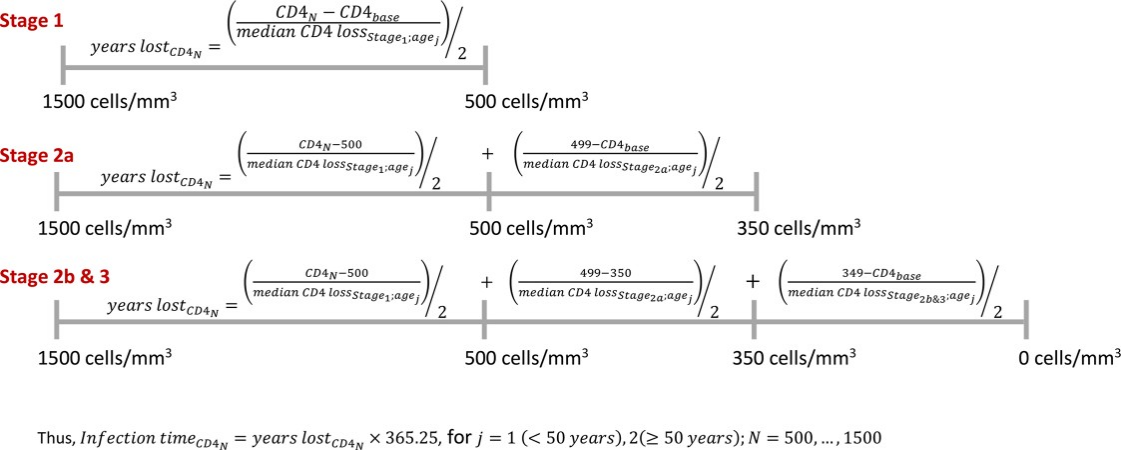
Formula to calculate the number of years lost from HIV sero-conversion and HIV diagnosis, and the time of infection, stratified by stage of HIV infection and age at diagnosis, assuming 500 to 1500 cells/mm3 as the normal range among uninfected individuals.

The main outcomes from this analysis were the duration of infection from HIV seroconversion until diagnosis as well as age at and year of seroconversion. These outcomes were estimated for each individual based on the statistical model for each age group and stage of HIV infection (six models in total) as follows:

- For each model, we estimated the CD4 cell loss per six months from HIV diagnosis until the start of ART. We calculated summary statistics, stratified by key variables, including medians and quartiles.
- Due to the population variation in CD4 cell count among HIV-negative individuals, we assumed 500 to 1500 cells/mm^3^ as the normal range [24, 35]. Thus, based on the CD4 value at diagnosis and for each CD4 value in the normal range, we calculated the duration of infection from HIV seroconversion until diagnosis as illustrated in Fig 1.
- Estimates for the year of and age at HIV seroconversion below the lower boundary (*i*.1980, which we assumed to be the year of the first possible infection case in Canada [36, 37]; or *ii*. minimum age of infection of 16 years [38, 39], as very few infections are acquired perinatally in BC and this is the minimum age of sexual consent to sexual activity in Canada) were discarded.
- Given that we had a different estimate for each of our outcomes for each value in the range 500 to 1500 cells/mm^3^, the final results were summarized by medians and quartiles.

### Ethics approval

This study was approved by the University of British Columbia ethics review committee at the St. Paul’s Hospital, Providence Health Care site (H18-02208 and H05-50123). As per the Research Ethics Board approval for this study and in compliance with relevant local legislation, informed consent was not required for this analysis, which was an approved secondary use of the administrative data involved.

## Results

Longitudinal data for a total of 4591 individuals who received ART for the first time in BC were considered. Of those, 403 (8.8%) individuals were removed as they were considered to be experiencing acute or recent HIV infection, 1066 (23.2%) for having no information on the stage of HIV infection at diagnosis, and 1869 (40.7%) were removed for not meeting the other eligibility criteria (1751 [38.1%] for having only one value for CD4, 117 [2.5%] for having missing information on CD4 and viral load, and one [0.02%] for having a CD4 out of range) (eTable 1 in S1 File). eTable 2 in S1 File presents the comparison between individuals included versus excluded from the study. Those included were more likely to be White, gbMSM, diagnosed between 2000 and 2003, with a higher CD4 cell count, without an AIDS diagnosis and a slightly lower viral load.

Thus, in total, 1253 (27.3%) individuals were included in this analysis with a total of 6542 CD4 measurements. The median number of CD4 measurements per individual, between HIV diagnosis and start of ART, was 5.0 (25^th^-75^th^ percentile [Q_1_-Q_3_], 3.0 to 9.0) (Table 1). Of these, 80.0% were male, 33.0% were White, 36.5% gbMSM, 39.0% PWID, 97.0% had no AIDS defining illness at HIV diagnosis and 59.6% were diagnosed before 2004. Additionally, at the time of HIV diagnosis, the median age was 38 (Q_1_-Q_3_, 31 to 45) years, CD4 cell count was 450 (Q_1_-Q_3_, 320 to 610) cells/mm^3^, viral load was 4.6 (Q_1_-Q_3_, 4.0 to 5.0), and these individuals were followed for a median of 2.1 (Q_1_-Q_3_, 1.1 to 4.0) years until ART initiation.

**Table 1:** Characteristics of eligible individuals at the time of HIV diagnosis.

Characteristics at Diagnosis	Study Population
**Sex, n(%)**	
Male	1002 (80.0)
Female	251 (20.0)
**Ethnicity, n(%)**	
Non-White	281 (22.4)
White	414 (33.0)
Unknown	558 (44.5)
**HIV Acquisition Risk Group, n(%)**	
gbMSM	457 (36.5)
PWID	489 (39.0)
gbMSM/PWID	103 (8.2)
Heterosexual	182 (14.5)
Other/Unknown	22 (1.8)
**AIDS Defining Illness, n(%)**	
No	1216 (97.0)
Yes	37 (3.0)
**Year of diagnosis, n(%)**	
<1996	67 (5.4)
1996–1999	264 (21.1)
2000–2003	415 (33.1)
2004–2007	251 (20.0)
2008–2013	256 (20.4)
**Age (years)**	
Median (Q_1_-Q_3_)	37.7 (31.2–45.1)
**CD4 cell count (cells/mm**^**3**^**)**	
Median (Q_1_-Q_3_)	450 (320–610)
**Viral load (log**_**10**_ **copies/mL)**	
Median (Q_1_-Q_3_)	4.6 (4.0–5.0)
**Follow-up time (years)**	
Median (Q_1_-Q_3_)	2.1 (1.1–4.0)
**Number of 6-month intervals for CD4 measurements**	
Median (Q_1_-Q_3_)	5.0 (3.0–9.0)

Abbreviations: gbMSM—gay, bisexual and other men who have sex with men; PWID—people who have ever injected drugs; Q_1_-Q_3_ - 25^th^-75^th^ percentiles.

The distribution of individuals in our strata was: 462 (36.9%) in Stage 1 and age <50 years; 64 (5.1%) in Stage 1 and age ≥50 years; 302 (24.2%) in Stage 2a and age <50 years; 51 (4.1%) in Stage 2a and age ≥50 years; 319 (25.5%) in Stages 2b&3 and age <50 years; and 54 (4.3%) in Stages 2b&3 and age ≥50 years. The CD4 cell trajectories for each stage of HIV infection and age stratum are shown in eFig1 in S1 File. The median number of CD4 measurements per individual, for each stratum, was 7.0 (Q_1_-Q_3_, 3.0 to 11.0), 5.0 (Q_1_-Q_3_, 3.0 to 7.0), 5.0 (Q_1_-Q_3_, 3.0 to 9.0), 4.0 (Q_1_-Q_3_, 3.0 to 7.0), 3.0 (Q_1_-Q_3_, 2.0 to 5.0) and 2.0 (Q_1_-Q_3_, 2.0 to 4.0), respectively.

**Fig 2:**
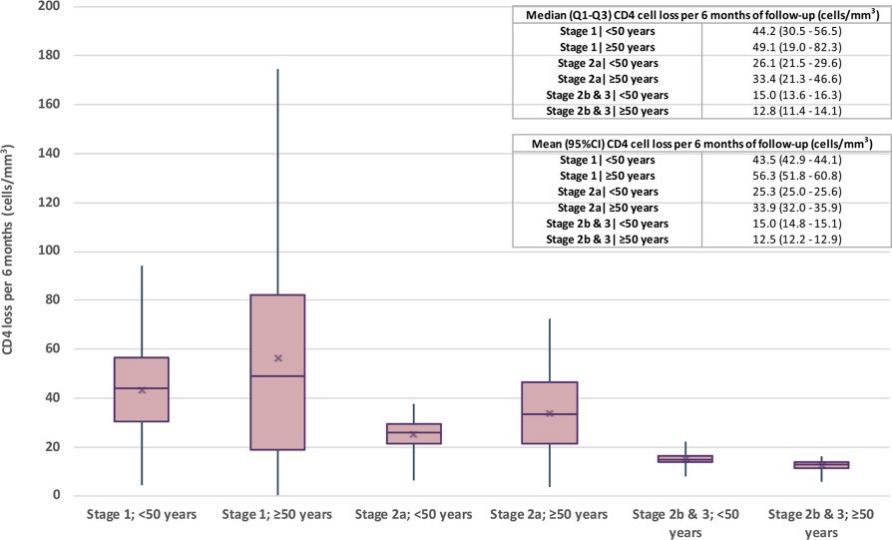
Estimated CD4 cell loss per six months from HIV diagnosis to start of antiretroviral therapy, stratified by stage of HIV infection and age at diagnosis.

The fitted models and goodness-of-fit assessments can be found in the Supplement. The estimated statistics for CD4 cell depletion per six months are shown in Fig 2. We observed that the median CD4 cell loss for each stratum was 44.2 (Q_1_-Q_3_, 30.5 to 56.5), 49.1 (Q_1_-Q_3_, 19.0 to 2.3), 26.1 (Q_1_-Q_3_, 21.5 to 29.6), 33.4 (Q_1_-Q_3_, 21.3 to 46.6), 15.0 (Q_1_-Q_3_, 13.6 to 16.3) and 12.8 (Q_1_-Q_3_, 11.4 to 14.1) cells/mm^3^, respectively. In Stages 1 and 2a, individuals aged ≥50 years lost CD4 cell counts faster than those <50 years, and this association was reversed in the last stages (2b & 3). Please note that the values for mean and associated 95% confidence interval were very similar.

**Fig 3:**
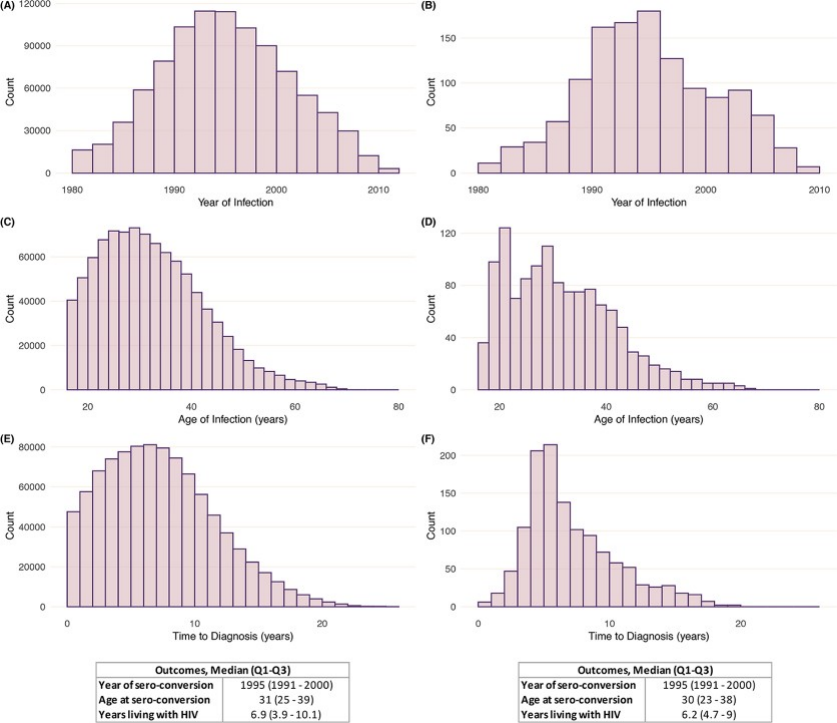
Estimates for the year of seroconversion (A-B), age at seroconversion (C-D), and duration of infection from HIV seroconversion until diagnosis (E-F). Note: For each outcome, the counts on the left are for the estimated values based on each CD4 value in the normal range for each individual (i.e., a maximum of 1000 observations per individual). For each outcome, the counts on the right are for the estimated individual-level values summarized as the median and quartiles of the corresponding distribution on the left (i.e., a maximum of 1 observation per individual).

**Fig 4:**
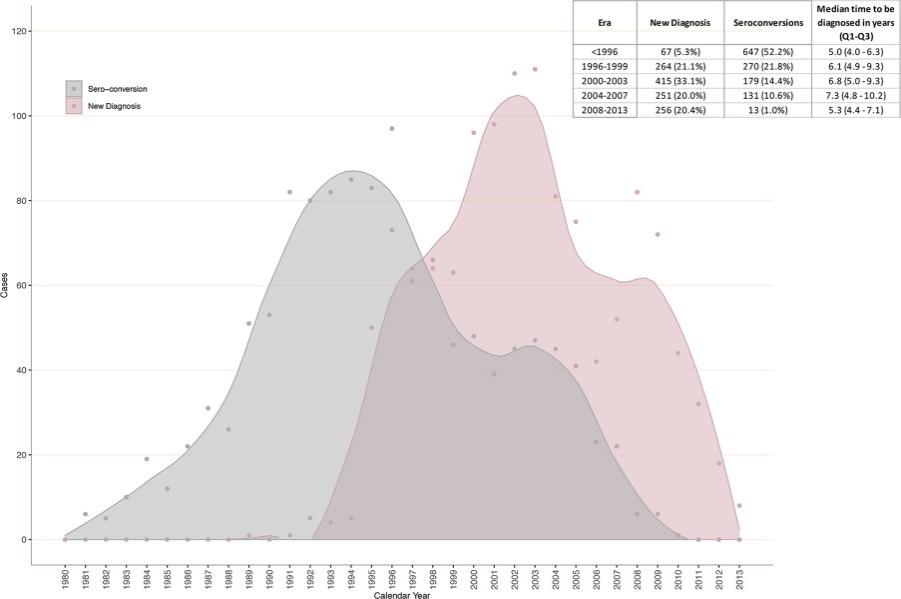
Comparison of the distribution of year of HIV diagnosis and estimated year of HIV seroconversion.

Thus, using the median CD4 depletion in each stratum, applying the formula in Fig 1, and assuming the normal CD4 range of 500 to 1500 cells/mm^3^, the results for duration of infection from HIV seroconversion until diagnosis as well as age at and year of HIV seroconversion are presented in Fig 3. Note that we could not estimate these outcomes for 13 individuals since their estimated values fell below the stated lower boundary. Based on the estimated outcomes for each CD4 value in the normal range, we obtained that the median year of seroconversion was 1995 (Q_1_-Q_3_, 1991 to 2000), age at seroconversion was 31 (Q_1_-Q_3_, 25 to 39) years and duration of infection from seroconversion until HIV diagnosis was 6.9 (Q_1_-Q_3_, 3.9 to 10.1) years. Those in Stages 1, 2a and 2b-3 were diagnosed after a median of 4.6 (Q_1_-Q_3_, 3.8 to 5.2), 6.9 (Q_1_-Q_3_, 6.1 to 7.7), and 11.0 (Q_1_-Q_3_, 9.3 to 13.4) years after the estimated HIV seroconversion date, respectively. In Fig 4, we present the comparison between the distribution of year of HIV diagnosis and estimated year of seroconversion. Compared with the annual new diagnoses, the peak of seroconversions occurred before 1996 versus 2000–2003 for new diagnoses. We also observed that the time to HIV diagnosis increased until the 2004–2007 period, and it decreased quite significantly from 2008 to 2013.

## Discussion

In this study, we demonstrated that a model for CD4 cell depletion could be used to estimate the duration of infection from HIV seroconversion until diagnosis, as well as age at and year of seroconversion. We also showed that in order to estimate these parameters, it is important to take into consideration the stage of HIV infection at diagnosis and different demographic, clinical and behavioural variables. The proposed methodology and parameters that we have estimated can be further used to estimate the date of seroconversion of PLWH, in similar settings and demographic profiles as the one in this study, without having to fit this model again. Another advantage of this method is the possibility of establishing uncertainty around the estimates. However, in order to use this methodology, we stress the importance of obtaining specific information at the time of a HIV diagnosis, especially CD4 cell count and age.

Based on our study, individuals aged ≥50 years in Stages 1 and 2a (CD4 ≥350 cells/mm^3^) lost CD4 faster than those <50 years, and this association was reversed in the Stages 2b & 3 (CD4 <350 cells/mm^3^). We believe this result was due to the smaller sample size for those aged ≥50 years in Stages 2b & 3. We also showed that the time to diagnosis varied significantly by year of estimated seroconversion, with a median ranging from 5.0 to 7.3 years before 2008 and 5.3 years thereafter. It is important to remember that during this time, there were key changes in HIV treatment guidelines, shortening the period of time an individual needs to wait between diagnosis and treatment initiation, which could explain these estimates. For example, in 2010 there was the launch of the STOP HIV/AIDS program in 2010, which aims to expand HIV testing, treatment, and support services to all PLWH in BC [2, 40].

As mentioned, our methodology and outputs can be further extrapolated to assist in the estimation of HIV prevalence, which is crucial to monitor the United Nations 90-90-90 Targets [41, 42]. These targets propose that, by 2020, at least 90% of PLWH should be diagnosed and aware of their HIV status; at least 90% of those diagnosed be on ART; and at least 90% of those on ART be virologically suppressed. If this target is reached, a 73% virologic suppression coverage will be achieved among all PLWH [41]. Estimating HIV prevalence, which depends on HIV incidence, is not trivial and different methods, depending on data availability, have been proposed. These methods include back-calculations [43–45], next-generation sequencing [46], prevalence surveys [47–49], mathematical modeling [50, 51], and CD4 cell count depletion-based approaches [7–11].

The Public Health Agency of Canada generates biennial national estimates of HIV incidence and prevalence, for the country and for each province and territory, utilizing sophisticated methodologies based on a back-calculation method [43]. Their method relies heavily on routine HIV/AIDS surveillance data (i.e., information on HIV testing, mortality, and recency of HIV infection). Unfortunately, this methodology does not consider demographic, clinical and behavioural heterogeneity that exists among individuals in a population. In addition, this methodology is not very sensitive to changes in policies regarding HIV testing and treatment initiation or the effect of ART in prolonging survival. Consequently, this method can yield estimates with substantial uncertainty, especially in more recent years [52, 53]. Thus, based on a broader population than the one in analyzed in our study, we can use the estimated parameters to reconstruct the HIV epidemic curve, and, ultimately, estimate the HIV prevalence and incidence over time. It would also be possible to estimate the percent of undiagnosed infections for the Canadian population (as well as for provinces and territories) as shown by Song *et al*. for the United States [7].

There are some potential limitations in this study. First, our estimates relied on individuals with at least two measurements of CD4 and viral load. Thus, we did not include individuals with one measurement nor did we use imputation methods to overcome this issue. However, by using the methodology in this study, which considers both intra- and inter-individual variation in the CD4 depletion trajectories (while also adjusting for key explanatory variables and providing goodness-of-fit assessments), we were able to fit robust models for CD4 cell depletion. Second, an important and complicated challenge in this type of analysis is the presence of right truncation of the CD4 data since we stopped following individuals when they initiated treatment. Thus, some individuals would have a shorter follow-up than others, and therefore, fewer CD4 cell count measurements. While mixed effects models largely address this issue, by applying a methodology such as the one by Liu *et al*. or Wu *et al*., we can further assess the robustness of our findings by examining biases that we may have in our analysis [54, 55]. Third, when estimating our outcomes, we discarded information below the lower boundary established for our estimates. This included assuming a minimum age of infection of 16 years, which excluded 1% of the data not being used to estimate the outcomes. In BC, between 1993 and 2017 there has been less than 40 perinatally acquired HIV infections [38]. In addition, although the minimum age at HIV diagnosis is between 15 and 19 years old, most HIV new diagnoses occurred after the age of 20 years [38]. Thus, although this restriction could have biased our results, we believe that this bias was minimal. Finally, our estimates for CD4 cell depletion considered ethnicity as a variable in the models, despite a large number of individuals in our study being classified as ethnicity unknown. Klein *et al*. showed that individuals of Black ethnicity had a slower rate of CD4 cell depletion that other ethnicities, even after controlling for HIV viral subtype [56]. In our database, only 23 (1.8%) individuals reported having Black ethnicity, and we do not expect that this number will be much higher due the historical data available during the study period.[38] Although, we could have left out this variable from our model, we let the variable selection method inform whether this variable should be included or not in our final model.

## Conclusions

Considering the heterogeneity that exists in individual CD4 cell trajectories in a population, we presented a methodology that only relies on routinely collected HIV-related data. This methodology yielded robust estimates that can be used in the future to retrospectively estimate other epidemic measures of morbidity, including the proportion of undiagnosed infections, that can be used to assess our progress towards HIV epidemic control in BC.

## Supporting information

S1 File (DOCX)
