# Supplementary material for: Estimation of time of HIV seroconversion using a modified CD4 depletion model
Source: PLoS One. 2021 Feb 12;16(2):e0246135. doi: 10.1371/journal.pone.0246135 (PMC7880448; doi:10.1371/journal.pone.0246135)
Supplement: S1 File — (DOCX) [file pone.0246135.s001.docx]

**Estimation of Time of HIV Seroconversion Using a Modified CD4 Depletion Model**

**Table of Contents**

[STOP HIV/AIDS Description 2](#_Toc57362871)

[eTable 1. Table describing the exclusion criteria in our study 4](#_Toc57362872)

[eTable 2. Comparison of individuals included and excluded from the study. 5](#_Toc57362873)

[Example for use of the formulas in Figure 1 to estimate the duration of infection from HIV seroconversion until diagnosis as well as age at seroconversion 6](#_Toc57362874)

[eFigure 1. CD4 cell trajectories from HIV diagnosis to start of antiretroviral therapy, stratified by stage of HIV infection and age at diagnosis. (A) Stage 1 and age ≥50 years; (B) Stage 1 and age <50 years; (C) Stage 2a and age ≥50 years; (D) Stage 2a and age <50 years; (E) Stages 2b&3 and age ≥50 years; (F) Stages 2b&3 and age <50 years. 7](#_Toc57362875)

[Brief Model Description and Goodness-of-fit 8](#_Toc57362876)

[References 10](#_Toc57362877)

# STOP HIV/AIDS Description

Data for the British Columbia Seek and Treat for Optimal Prevention of HIV/AIDS (STOP HIV/AIDS) population-based cohort was derived from various linkages between provincial administrative databases.

**Data Steward: British Columbia Centre for Disease Control**^1,2^

1. Provincial HIV/AIDS Surveillance Database: a surveillance database that collates all HIV laboratory testing, new HIV diagnosis and occurrence of AIDS-defining illnesses data;

**Data Steward: British Columbia Centre for Excellence in HIV/AIDS**^3,4^

1. Drug Treatment and Laboratory Databases, which captures all antiretroviral dispensing data, plasma viral load testing, drug resistance testing, occurrence of AIDS-defining illnesses, approximately 85% of CD4 cell count measurements, and key patient demographic information;

**Data Steward: British Columbia Ministry of Health**^5^

1. The Medical Services Plan (MSP) billing database, which captures HIV and non-HIV-related inpatient and outpatient services provided by physicians and supplementary health care practitioners, as well as diagnostic procedures. This database also contains cost associated with claims paid through fee-for-service and the Alternative Payment Program;
2. Home and Community Care database, which captures a variety of services including hospice and home nursing care, adult day services, assisted living, respite care, residential and convalescent care
3. Mental Health Services database, which captures utilization of mental health services including fee-for-service, institutional care, community clinics and acute care.
4. Addictions Information Management Systems, which captures referral to treatment for alcohol, drug or gambling addictions.
5. The PharmaNet database is a real-time system, which captures all prescriptions for drugs and medical supplies dispensed from community pharmacies in BC as well as prescriptions dispensed from hospital outpatient pharmacies use at home. Note that this database does not capture antiretroviral dispensing data;
6. The Client Roster or Consolidation File, which captures individual demographic and geographic data. This database is also used to construct population denominators.

**Data Steward: Canadian Institute for Health Information**^6^

1. The Discharge Abstract Database (DAD), which captures all discharges, transfers and deaths of in-patients and day surgery patients from acute care hospitals across BC;

**Data Steward: British Columbia Vital Statistics Agency**^7^

1. The Vital Statistics database, which records death information of all BC’s residents.

# eTable 1. Table describing the exclusion criteria in our study

| **Exclusion criteria** | **Number of Individuals** | |
| --- | --- | --- |
|  | **Included** | **Excluded** |
| Eligible individuals were aged ≥18 years at HIV diagnosis, which happened between 1989 and 2013. These individuals started antiretroviral therapy for the first time in British Columbia between 1996 and 2015.  Both antiretroviral therapy initiation date and HIV diagnosis date are not missing. | 4591 | - |
|  |  |  |
|  |  |  |
| Removed individuals considered to be experiencing acute or recent HIV infection | 4188 | 403 |
| Removed individuals without information on the stage of HIV infection at diagnosis | 3122 | 1066 |
| Removed individuals with a CD4 cell count outside the normal range | 3121 | <5 |
| Removed individuals with missing information on CD4 cell count and viral load | 3004 | 117 |
| Removed individuals for having only one value for CD4, that is, without a follow-up record after HIV diagnosis | 1253 | 1751 |

eTable 2. Comparison of individuals included and excluded from the study.

| **Characteristics at Diagnosis** | **Included** | | **Excluded** | | **P-value*** |
| --- | --- | --- | --- | --- | --- |
|  | **(N=1253)** | | **(N=3338)** | |  |
|  | **N** | **(%)** | **N** | **(%)** |  |
| **Sex** |  |  |  |  | 0.2050 |
| Male | 1002 | (27.7) | 2612 | (72.3) |  |
| Female | 251 | (25.7) | 726 | (74.3) |  |
| **Ethnicity** |  |  |  |  | 0.0200 |
| Non-White | 281 | (27.5) | 739 | (72.5) |  |
| White | 414 | (29.8) | 974 | (70.2) |  |
| Unknown | 558 | (25.6) | 1625 | (74.4) |  |
| **HIV Acquisition Risk Group** |  |  |  |  | <0.0001 |
| gbMSM | 457 | (29.3) | 1105 | (70.7) |  |
| PWID | 489 | (28.9) | 1202 | (71.1) |  |
| gbMSM/PWID | 103 | (27.0) | 278 | (73.0) |  |
| Heterosexual/Other | 182 | (21.0) | 686 | (79.0) |  |
| Unknown | 22 | (24.7) | 67 | (75.3) |  |
| **AIDS Defining Illness** |  |  |  |  | <0.0001 |
| No | 1216 | (29.2) | 2949 | (70.8) |  |
| Yes | 37 | (8.7) | 389 | (91.3) |  |
| **Year of diagnosis** |  |  |  |  | <0.0001 |
| <1996 | 67 | (26.0) | 191 | (74.0) |  |
| 1996-1999 | 264 | (25.8) | 758 | (74.2) |  |
| 2000-2003 | 415 | (37.8) | 682 | (62.2) |  |
| 2004-2007 | 251 | (23.8) | 805 | (76.2) |  |
| 2008-2013 | 256 | (22.1) | 902 | (77.9) |  |
| **Continuous Variable** | **Median** | **(Q1-Q3)** | **Median** | **(Q1-Q3)** |  |
| **Age (years)** | 37.7 | (31.2-45.1) | 38.1 | (30.9-45.9) | 0.3251 |
| **CD4 cell count (cells/mm^3^)** | 450 | (320-610) | 220 | (70-420) | <0.0001 |
| **Viral load (log_10_ copies/mL)** | 4.6 | (4.0-5.0) | 5.0 | (4.4-5.0) | <0.0001 |

Abbreviations: gbMSM - gay, bisexual and other men who have sex with men; PWID - people who have ever injected drugs; Q1-Q3 - 25th-75th percentiles.

*Bivariable comparisons of categorical variables were done by the chi-square test; Bivariable comparisons of continuous variables were done by the Wilcoxon's rank sum test.

# Example for use of the formulas in Figure 1 to estimate the duration of infection from HIV seroconversion until diagnosis as well as age at seroconversion


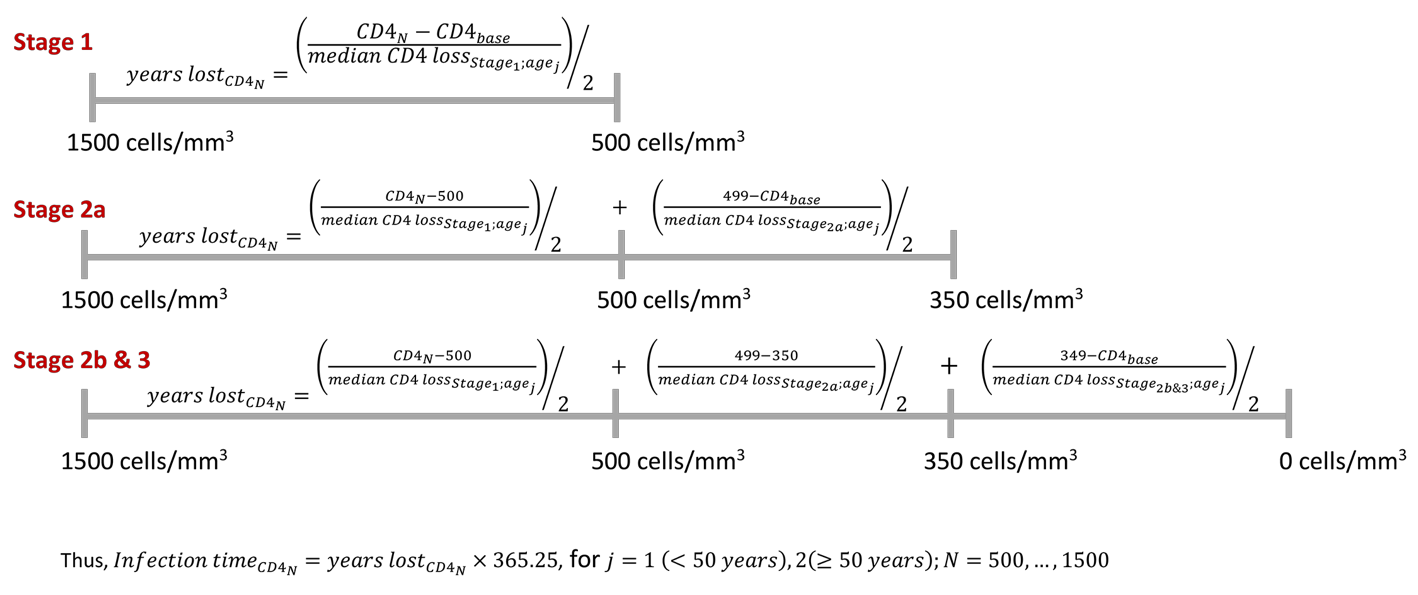


Due to the population variation in CD4 cell count among HIV-negative individuals, we assumed 500 to 1500 cells/mm^3^ as the normal range. Thus, based on the CD4 value at diagnosis and for each CD4 value in the normal range, we calculated the duration of infection from HIV seroconversion until diagnosis.

**First**, we show the calculations for individuals diagnosed in Stage 1 (CD4 cell count ≥500 cells/mm^3^), aged <50 years, using the median CD4 cell loss per 6 months of follow-up as in Figure 2.

Suppose an individual has a CD4 value at diagnosis of 1200 cells/mm^3^, measured on June 1, 2008.

Starting with the maximum CD4 in the normal range being 1500 cells/mm^3^, then:

${years lost}_{{CD4}_{1500}}=\frac{\left( \frac{1500-{CD4}_{base}}{{median CD4 loss}_{{Stage}_{1};age <50}} \right)}{2}=\frac{\left( \frac{1500-1200}{44.2} \right)}{2}=3.39$

${Infection time}_{{CD4}_{1500}}={Date of {CD4}_{base}- years lost}_{{CD4}_{1500}}\times365.25$= January 9, 2005

Since we do not know what the normal CD4 is for this person, we will need to do the same calculation for each CD4 value in normal range until we reach 1201 cells/mm^3^. We will repeat this process for everyone in this group.

**Second**, we show the calculations for individuals in Stage 2a (CD4 from 350 to 499 cells/mm^3^), aged <50 years, using the median CD4 cell loss per 6 months of follow-up as in Figure 2.

Suppose an individual has a CD4 value at diagnosis of 400 cells/mm^3^, measured on May 1, 2012.

Starting with the maximum CD4 in the normal range being 1500 cells/mm^3^, then:

$${years lost}_{{CD4}_{1500}}=\frac{\left( \frac{1500-500}{{median CD4 loss}_{{Stage}_{1};age <50}} \right)}{2}\frac{+\left( \frac{499-{CD4}_{base}}{{median CD4 loss}_{{Stage}_{1};age <50}} \right)}{2}=$$

$= \frac{\left( \frac{1500-500}{44.2} \right)}{2}+\frac{\left( \frac{499-400}{26.1} \right)}{2}=13.21$

${Infection time}_{{CD4}_{1500}}={Date of {CD4}_{base}- years lost}_{{CD4}_{1500}}\times365.25$= February 14, 1999

Since we do not know what the normal CD4 is for this person, we will need to do the same calculation for each CD4 value in normal range until we reach 500 cells/mm^3^. We will repeat this process for everyone in this group.

**Third**, we show the calculations for individuals in Stage 2a (CD4 from <350 cells/mm^3^), aged <50 years, using the median CD4 cell loss per 6 months of follow-up as in Figure 2.

Suppose an individual has a CD4 value at diagnosis of 300 cells/mm^3^, measured on August 1, 2011.

Starting with the maximum CD4 in the normal range being 1500 cells/mm^3^, then:

$${years lost}_{{CD4}_{1500}}=\frac{\left( \frac{1500-500}{{median CD4 loss}_{{Stage}_{1};age <50}} \right)}{2}\frac{+\left( \frac{499-350}{{median CD4 loss}_{{Stage}_{2a};age <50}} \right)}{2}\frac{+ \left( \frac{349-{CD4}_{base}}{{median CD4 loss}_{{Stage}_{2b\&3};age <50}} \right)}{2}$$

$$=\frac{\left( \frac{1500-500}{44.2} \right)}{2}\frac{+\left( \frac{499-350}{26.1} \right)}{2}\frac{+ \left( \frac{349-300}{15.0} \right)}{2}=15.80$$

${Infection time}_{{CD4}_{N}}={Date of {CD4}_{base}- years lost}_{{CD4}_{N}}\times365.25$= October 13, 1995

Once more, since we do not know what the normal CD4 is for this person, we will need to do the same calculation for each CD4 value in normal range until we reach 500 cells/mm^3^. We will repeat this process for everyone in this group.

**Finally,** once we run this process for each individual in the study, we merged the data for the entire study population, and then calculated the median, 25^th^ and 75^th^ percentiles of the values that we estimated through the calculations above. Based on the date of birth and the estimated infection time, we estimated the age at sero-conversion.

# eFigure 1. CD4 cell trajectories from HIV diagnosis to start of antiretroviral therapy, stratified by stage of HIV infection and age at diagnosis. (A) Stage 1 and age ≥50 years; (B) Stage 1 and age <50 years; (C) Stage 2a and age ≥50 years; (D) Stage 2a and age <50 years; (E) Stages 2b&3 and age ≥50 years; (F) Stages 2b&3 and age <50 years.

# Brief Model Description and Goodness-of-fit

**Stage 1 and age <50 years**

Nonlinear mixed-effects model adjusted for time, viral load, sex, year of HIV diagnosis, ethnicity, follow-up time and AIDS at HIV diagnosis.

Random effects: intercept and slope for the longitudinal viral load measurements





**
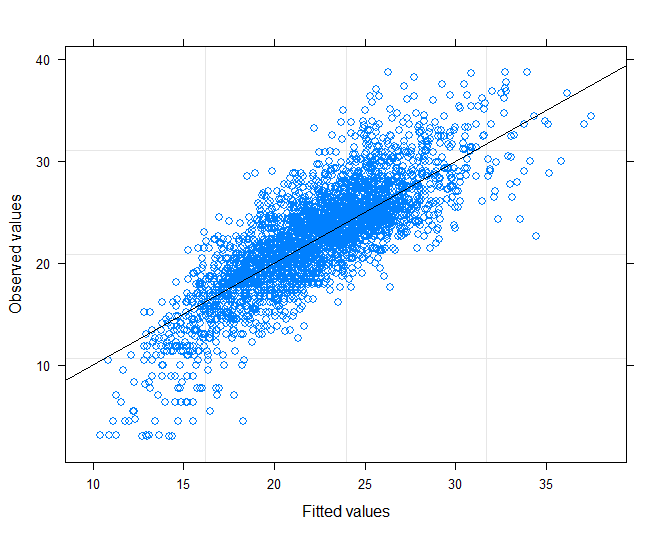
Stage 1 and age ≥50 years**

Nonlinear mixed-effects model adjusted for time, viral load, and follow-up time.

Random effects: intercept





**Stage 2a and age <50 years**

Nonlinear mixed-effects model adjusted for time, viral load, sex, year of HIV diagnosis, HIV transmission risk group, and follow-up time.

Random effects: intercept and slope for the longitudinal viral load measurements





**Stage 2a and age ≥50 years**

Nonlinear mixed-effects model adjusted for time, year of HIV diagnosis, ethnicity, and follow-up time.

Random effects: intercept





**Stages 2b & 3 and age <50 years**

Linear mixed-effects model adjusted for time, viral load, AIDS at HIV diagnosis, year of HIV diagnosis, HIV transmission risk group, and follow-up time. Note that the exponential decay function was not needed as the measurements were quite liner over time (see eFigure 1).

Random effects: intercept





**Stages 2b & 3 and age ≥50 years**

Linear mixed-effects model adjusted for time, viral load, AIDS at HIV diagnosis, ethnicity, and HIV transmission risk group. Note that the exponential decay function was not needed as the measurements were quite liner over time (see eFigure 1).

Random effects: intercept

**

**

# References

1. British Columbia Centre for Disease Control. HIV/AIDS Information System (HAISYS). Clinical Prevention Services, British Columbia Centre for Disease Control, 2016. <http://www.bccdc.ca/about/accountability/data-access-requests/public-health-data>.

2. British Columbia Centre for Disease Control Public Health Laboratory. HIV laboratory testing datasets (tests: ELISA, Western blot, NAAT, p24, culture). Clinical Prevention Services, British Columbia Centre for Disease Control, 2016. <http://www.bccdc.ca/about/accountability/data-access-requests/public-health-data>.

3. Patterson S, Cescon A, Samji H, et al. Cohort Profile: HAART Observational Medical Evaluation and Research (HOMER) cohort. *Int J Epidemiol.* 2015;44(1):58-67.

4. British Columbia Centre for Excellence in HIV/AIDS (BCCfE). Drug Treatment Program. 2019; <http://cfenet.ubc.ca/drug-treatment-program>. Accessed 9 October 2019.

5. British Columbia Ministry of Health [**creator**] (2016):. Medical Services Plan (MSP) Payment Information File; Consolidation File (MSP Registration & Premium Billing); Home & Community Care (Continuing Care); Mental Health; PharmaNet. British Columbia Ministry of Health [**publisher**]. Data Extract. MOH (2016). <http://www2.gov.bc.ca/gov/content/health/conducting-health-research-evaluation/data-access-health-data-central>.

6. Canadian Institute of Health Information [**creator**] (2016):. Discharge Abstract Database (Hospital Separations). British Columbia Ministry of Health [**publisher**]. Data Extract. MOH (2016). <http://www2.gov.bc.ca/gov/content/health/conducting-health-research-evaluation/data-access-health-data-central>.

7. British Columbia Vital Statistics Agency [**creator**] (2016):. Vital Statistics. British Columbia Ministry of Health [**publisher**]. Data Extract. MOH (2016). <http://www2.gov.bc.ca/gov/content/health/conducting-health-research-evaluation/data-access-health-data-central>.
